# Bioavailability and Brain-Targeting of Geniposide in Gardenia-Borneol Co-Compound by Different Administration Routes in Mice

**DOI:** 10.3390/ijms131114127

**Published:** 2012-11-01

**Authors:** Yang Lu, Shouying Du, Jie Bai, Pengyue Li, Ran Wen, Xuejiao Zhao

**Affiliations:** School of Chinese Materia Medica, Beijing University of Chinese Medicine, No. 6, Zhonghuan South Road, Wangjing, Chaoyang District, Beijing 100102, China; E-Mails: landocean28@163.com (Y.L.); baijie22811@163.com (J.B.); pengyuelee@126.com (P.L.); ran09291210@sina.com (R.W.); zxj20100931426@163.com (X.Z.)

**Keywords:** geniposide, borneol, intranasal, intragastric, intravenous, pharmacokinetics, brain-target

## Abstract

Both geniposide (Ge) and borneol (Bo) are bioactive substances derived from traditional Chinese medicine. Injections containing co-compound of Gardenia-Borneol are widely used for stroke treatment in China, such as “Xingnaojing” multi-component injection. As more and more adverse reactions (especially drug allergy) were reported, it is urgent to find more effective and safer routes of administration for such kinds of medicines. In this paper, bioavailabilities and brain-target effects of geniposide in Gardenia-Borneol co-compound through different administration routes in mice were investigated. Geniposide concentrations in plasma and in brain of mice were determined by reversed-phase high-performance liquid chromatography. The pharmacokinetics parameters of intranasal (i.n.) and intragastric (i.g.) administration were compared with intravenous (i.v.) administration. The bioavailabilities of Ge were 85.38% and 28.76% for i.n. and i.g. while *T*_max_ were 1 min and 30 min. *C*_max_ were 21.881 ± 5.398, 1.914 ± 0.327 and 42.410 ± 6.268 μg/mL for i.n., i.g. and i.v., respectively. The AUC of Ge in brain were 32413.6 ± 4573.9, 6440.1 ± 863.7 and 37270.5 ± 4160.6 ng/g·min for i.n., i.g. and i.v., respectively. The drug target indexes (DTI) were 1.02 and 0.60 for i.n. and i.g. The results demonstrated that geniposide could be absorbed promptly and thoroughly by i.n. administration in mice and basically transported into the brain though blood vessel passways.

## 1. Introduction

**Figure 1:**
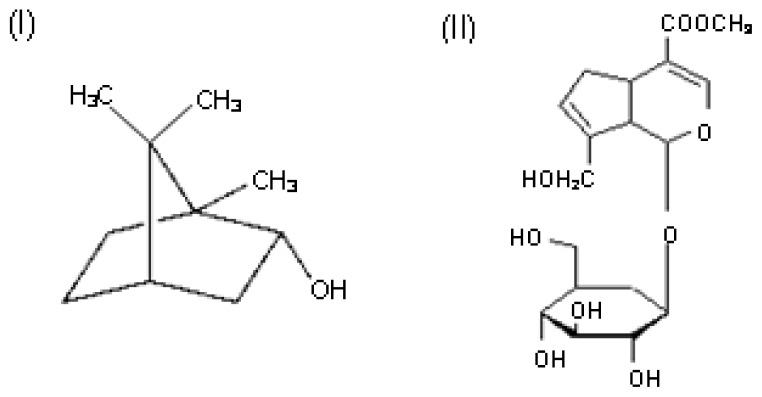
Structures of Bo (**I**) and Ge (**II**).

Geniposide (Ge), one of the iridoid glycoside extracted from gardenia fruit (*Gardenia jasminoides* Ellis, Rubiaceae), is widely used with Borneol (Bo) in the treatment of cerebrovascular and cardiovascular diseases in China for its anti-thrombotic and anti-inflammatory effects [1,2]. Bo is a monoterpenoid component in *Blumea balsamifera* (L.) DC. It is widely used in traditional Chinese medicine (TCM) combined with gardenia for stroke treatment, such as “Xingnaojing” injection. Our study [3,4] and some reports had shown that borneol could improve the nasal, oral and gastrointestinal bio-availability of drugs, accelerate the opening of the blood-brain-barrier (BBB) and enhance the distribution of drugs in brain tissue [5,6]. The structures of geniposide and borneol are shown in Figure 1.

The traditional Chinese medicine injections are derived from herbs. The macromolecular protein, fatty acid and other materials in these herb extracts can easily cause adverse reactions (especially drug allergy) through intravenous injection. The clinical main manifestations of XNJ injection adverse side effects were respiration system disorder at 33.0%, cardiology system disorder at 17.0%, lesion of skin and appendants at 13.2%, whole body disorder at 12.3%, sympathetic and parasympathetic nervous system disorders at 12.3%, central and peripheral nervous system disorders at 6.6%, gastro-intestinal system disorders at 3.8% and other types at 1.9%, according to the reference [7]. As more and more adverse reactions (especially drug allergy) have been reported on multi-component injection in China, it is urgent to find a safer route for traditional Chinese medical compounds, which has high bioavailability, quick absorption rate and good brain-targeting for cerebrovascular and cardiovascular disease treatment. The intranasal route could be a hopeful substitution, because drugs can be absorbed sufficiently and rapidly into blood [8,9] and can be transported from the nasal cavity directly into the central nervous system [10]. I.n. delivery is non-invasive, essentially painless, does not require sterile preparation and is easily and readily administered by the patient or a physician, e.g., in an emergency setting. For its non-invasiveness, the incidence of drug allergy by i.n. administration is lower than i.v., especially for Compound Chinese medicine.

In order to choose the best administration route of Xingnaojing (based on the famous traditional Chinese medicine prescription “AnGongNiuHuangWan”, mainly containing borneol and gardenia extract) in an emergency setting, we investigated bioavailabilities and brain-target effects of Ge in Gardenia-Borneol co-compound through different administration routes (intragastric, intranasal and intravenous) in mice. The concentrations of Ge in mouse plasma and brain after different administrations of Gardenia-Borneol co-compound were detected, and the pharmacokinetic parameters of each group were compared.

## 2. Results and Discussion

### 2.1. Method of Plasma Samples Qualification

**Figure 2:**
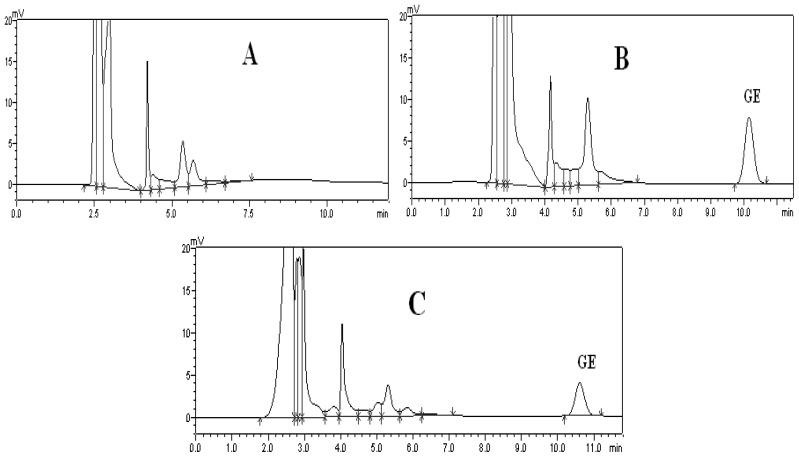
Typical HPLC chromatograms of blank mouse plasma (**A**); blank mouse plasma spiked with Ge (**B**); and plasma sample of a dosed mouse (**C**).

The HPLC method for assay of Ge in the plasma was specific and efficient. There was no interference from endogenous components observed at the retention times of all the analytes in the chromatograms. Analytical time was less than 12 min. Typical chromatograms of blank mouse plasma, blank mouse plasma spiked with Ge and mouse plasma sample are shown in Figure 2.

Plasma standards covering the expected sample concentration range were prepared by spiking various quantities of Ge into blank plasma. These calibration standards were used to validate the linearity, recovery and precision of the analytical method. The limit of detection of Ge was 25 ng/mL in plasma (S/N ≥ 3). The calibration curves of Ge were linear in the range of 0.27~21.6 μg/mL. There was a good linearity between *C* and *A* (*C* = 3.6494 × 10^−5^*A* − 0.0308, *r* = 0.99997). The accuracy of Ge at low, middle and high concentration was 96.74 ± 1.40%, 98.30 ± 1.78% and 99.48 ± 2.91%, respectively. The RSD of intra-day precision of Ge at low, middle and high concentrations were 2.00%, 1.27% and 0.38%, respectively, while inter-day precision were 4.09%, 0.33% and 1.08%. The plasma sample assay method was qualified and fit for our purposes.

### 2.2. Method of Brain Samples Qualification

**Figure 3:**
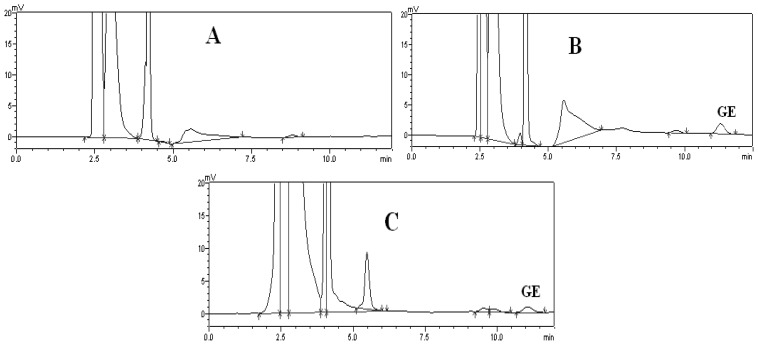
Typical HPLC chromatograms of blank brain homogenate of mouse (**A**); blank brain homogenate of mouse spiked with Ge (**B**); and brain homogenate sample of a dosed mouse (**C**).

The HPLC method for assay of Ge in the brain was specific and efficient. There was no interference from endogenous components observed at the retention times of all the analytes in the chromatograms. Analytical time was less than 12 min. Typical chromatograms of blank brain homogenate of mouse, blank brain homogenate of mouse spiked with Ge and mouse brain homogenate sample are shown in Figure 3.

Brain standards covering the expected sample concentration range were prepared by spiking various quantities of Ge into blank brain homogenate. These calibration standards were used to validate the linearity, recovery and precision of the analytical method. The limit of detection of Ge was 10 ng/g in brain (S/N ≥ 3). The calibration curves of Ge in brain were linear in the range of 54~1620 ng/g. There was a good linearity between *C* and *A* (*C* = 2.773 × 10^−2^*A* − 22.642, *r* = 0.9991). The mean relative recoveries of Ge in brain at low, middle and high concentrations were 98.72% ± 4.56%, 104.13% ± 4.68% and 97.15% ± 3.28%, respectively. The RSD of intra-day precision of Ge at low, middle and high concentrations were 1.67%, 2.02% and 0.71%, respectively, while inter-day precision were 4.09%, 2.11% and 2.80%. The brain sample assay method was qualified and fit for our purposes.

### 2.3. Statistical Analysis and Pharmacokinetics

**Figure 4:**
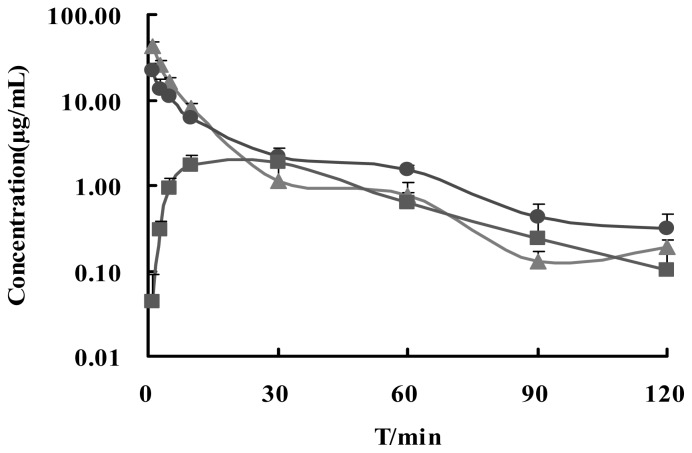
Mean plasma concentration-time curve of Ge (18 mg/kg of gardenia extract combined with 18 mg/kg of borneol) via i.n. (●); i.v. (▲) and i.g. (■) administration in mice (*n* = 5, mean ± S.D.).

The concentration-time profiles of Ge in plasma after i.v., i.n. and i.g. administrations are shown in Figure 4. Non-compartmental analysis of pharmacokinetic data was performed by Kinetica 4.4 software. The pharmacokinetic parameters are shown in Table 1. The absolute bioavailability was *F*, *F* = AUC_blood (i.n./i.g.)_/AUC_blood (i.v.)_ × 100%. The results showed that the average value of *C*_max_ in plasma was 42.410 ± 6.268, 21.881 ± 5.398 and 1.914 ± 0.327 μg/mL, AUC_0–120_ were 324.88 ± 37.62, 277.39 ± 22.65 and 93.44 ± 9.7 μg/mL·min for i.v., i.n. and i.g., respectively. The bioavailabilities of Ge were 85.38% and 28.76% for i.n. and i.g, while *T*_max_ were 1 min and 30 min. Our previous research demonstrated that nasal absorption of Ge can be enhanced effectively by Bo [4]. This result is consistent with our previous research that Ge in Gardenia-Borneol co-compound could be absorbed promptly and thoroughly by i.n. administration in mice, while the gastrointestinal absorption of Ge was inadequate and slow (*t*_1/2_ of absorption = 9.5 h) [11].

**Figure 5:**
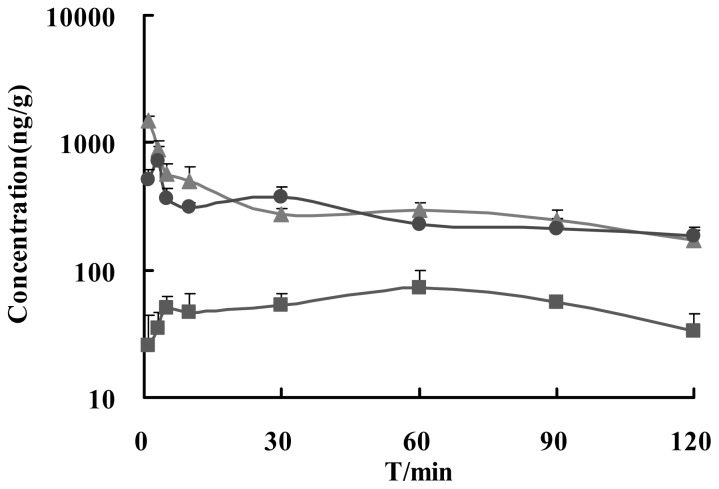
Mean brain concentration-time curve of Ge (18 mg/kg of gardenia extract combined with 18 mg/kg of borneol) via i.n. (●); i.v. (▲) and i.g. (■) administration in mice (*n* = 5, mean ± S.D.).

The concentration-time profiles of Ge in brain after i.v., i.n. and i.g. administrations are shown in Figure 5. Non-compartmental analysis of pharmacokinetic data was performed by Kinetica 4.4 as shown in Table 2. The relative brain targeted coefficient was *R*e, *R*e = AUC_brain (i.n./i.g.)_/AUC_brain (i.v.)_ × 100%. The brain/blood drug ratio was *T*e, *T*e = AUC_brain_/AUC_blood_ × 100%. So the drug target index was DTI, DTI = *T*e_(i.n. /i.g.)_/*T*e_(i.v)_. The results showed that the average value of *C*_max_ in brain was 1476.4 ± 145.1, 746.7 ± 174.8 and 76.2 ± 22.1 ng/g, while *T*_max_ was 1, 3 and 60 min for i.v., i.n. and i.g., respectively. AUC_0–120_ was 37270.5 ± 4160.6, 32413.6 ± 4573.9 and 6440.1 ± 863.7 μg/mL·min for i.v., i.n. and i.g., respectively. Therefore, Re was 86.97% and 17.28%, while DTI 1.02 and 0.60 for i.n. and i.g. compared to i.v. The results indicated that Ge in Gardenia-Borneol co-compound could be promptly and thoroughly transported into brain by i.n. The brain amount and distribution speed of Ge by i.n. was almost the same as i.v., while i.g. was poor and low.

## 3. Experimental

### 3.1. Chemicals, Reagents and Animals

Gardenia extract was prepared and purified by researchers (Ge, 60.8% purity). Ge was obtained from the National Institute for the Control of Pharmaceutical and Biological Products (NICPBP, Beijing, China). Borneol was purchased from Guizhou golden Pharmaceutical Co., Ltd. Acetonitrile and Methanol were of high performance liquid chromatography (HPLC) grade (Merck, Germany). Pure water (Wahaha, Hangzhou, China) was purchased from the market. The other chemical reagents were of analytical grade. ICR mice (male, weight 20–25 g, Certification No.0153199) were purchased from Weitong biotechnology Inc. (Beijing, China). All animals were clinically healthy and biochemically normal throughout the experimental period. The animals were fasted for 12 h with free access to water prior to treatment. All experimental procedures were conducted in accordance with the European Union guidelines for the use of experimental animals and approved by the Beijing University of Chinese Medicine Committee on Animal Care and Use.

### 3.2. Instrumentation

Instruments used in this study were a centrifuge (Anke TGL-16G, Anting Scientific Instrument Company, Shanghai, China); a vortex mixer (VDRTEX-5, Shanghai Medical University Instrument Company, Shanghai, China); and an analytical balance (METTLER-AE240). A LC-20AT HPLC system with UV detector (Shimadzu Company, Japan) was also used in the study.

### 3.3. Chromatographic Conditions

The separation was carried out using a Diamonsil C_18_ column (250 × 4.6 μm, i.d., 5.0 μm) under the following chromatographic conditions: column temperature, 25 °C; sample injection volume, 20 μL; flow rate, 1.0 mL/min; detection wavelength, 238 nm; and mobile phase, acetonitrile-water(14:86).

### 3.4. Sample Preparation

#### 3.4.1. Solution Preparation

1% (*v*/*v*) of Tween-80 and 20% (*v*/*v*) ethanol were dissolved in physiological saline and used as the solvent for drugs. 1.2 g of borneol fine powder and 1.2 g gardenia extract were dissolved into 10 mL solvent, named as co-compound nasal drops. Dilute 0.3 mL of nasal drops precisely with 1% Tween 80-normal saline solution into 10 mL, filtrate by 0.22 μm filter, named as co-compound injection. Dilute 0.3 mL of nasal drops precisely with 1% Tween 80-normal saline solution into 10 mL, named as co-compound intragastric solution.

#### 3.4.2. *In Vivo* Experiments

120 mice were divided into 3 groups (A, B, C), equally and randomly, 40 for each group. Group A was injected 0.1 mL co-compound injection via the tail vein. Group B was treated 3 μL nasal drops with a soft PE polyethylene tube after anesthesia by ether steam. Group C was treated 0.1 mL co-compound intragastric solution. The doses chosen for the mice were 18mg/kg·d (borneol and gardenia extract, respectively), 3 times based on the doses for human beings. The blood samples were collected into heparinized tubes by pricking the eyeball. Then, mice were killed by decollation. The corresponding brains were also collected at 1, 3, 5, 10, 30, 60, 90 and 120 min after drug administration. The brains were accurately weighed, and plasma was obtained after centrifugation. The sample processing and determination were immediately made when plasma and brain tissue were obtained.

Plasma sample (100 μL) was placed into a centrifuge tube. To precipitate proteins in the sample, 300 μL acetonitrile was added and vortexed for 1 min. After centrifugation at 10,000 rpm for 10 min, supernatant was removed and evaporated under air stream in a water bath at 60 °C. The residue was dissolved in 100 μL of methanol and vortexed for 30 s, centrifuged at 10,000 rpm for 10 min. 20 μL of supernatant was injected into the HPLC system for analysis.

The brains were washed by normal saline to remove excess surface blood and were dried by filter paper. The bloodless brains were homogenized after mixing with normal saline solution 1.5 times to the weight. After centrifugation at 12,000 rpm for 5 min, 600 μL methanol (the brain homogenate was 300 μL) was added in the supernatant to precipitate proteins in the sample. After being vortexed for 1 min, the solution was centrifuged at 12,000 rpm for 5 min. Then, supernatant was removed and evaporated under air stream in a water bath at 60 °C. The residue was dissolved in 100 μL of methanol and vortexed for 30 s, centrifuged at 12,000 rpm for 5 min. 20 μL aliquot supernatant was injected into the HPLC system for analysis.

### 3.5. Statistical Evaluation

The plasma and brain pharmacokinetic parameters of i.n., i.g. and i.v. groups were compared by one-way ANOVA at the 0.05 significance level, and the statistical differences were carried out by paired *t*-tests (Statistics Analysis System 8.0).

## 4. Conclusions

As TCM injections are used widely in China, more and more adverse effects have been reported. Thus, it is important to find a better way for TCM usage, especially for treating neurological diseases. Intranasal delivery is a noninvasive and convenient method that rapidly targets therapeutics to the central nervous system, bypassing the blood-brain barrier. In our formulation, Ge is a water-soluble compound with low molecular weight (388.4). Its log *p* value in octanol/water system was −0.97, suggesting that it was hard for Ge to permeate the mucous membrane. Its intranasal absorption coefficient (*K*) was 0.061 h^−1^ and 0.11 h^−1^ in Gardenia-Borneol co-compound [3]. When passing through the mucous membrane, Bo might enhance epithelial junction permeability and promote absorption of Ge in rats. The enhancing effect is temporary and closely related to the dose of Bo in rats [4].

In this research, the *C*_max_ of Ge in plasma and brain were highest via i.v. administration and has no absorption process. *C*_max_ of i.n. in plasma was up to 1/2 of the i.v., while i.g. was equivalent to 1/20. And yet, brain peak concentration of i.n. and i.g. was up to 1/2 and 1/20 compared to the i.v. group. *T*_max_ of Ge in plasma and brain of i.n. were 1 and 3 min, while *T*_max_ of i.g. were about 30 and 60 min, showing that the absorption rate of Ge in the nasal cavity of mice was higher than i.g., either in the blood or brain. The AUC_plasma_ of i.v., i.n. and i.g. were 324.88 ± 37.62, 277.39 ± 22.65 and 93.44 ± 9.71 μg/mL·min, respectively, while AUC_brain_ values were 37270.5 ± 4160.6, 32413.6 ± 4573.9 and 6440.1 ± 863.7 ng/g·min. The concentration (*C*_max_) and amount (AUC) of Ge in brain of i.n. group were in proportion with the concentration (*C*_max_) and amount of Ge in plasma, like the i.v. group. In the i.v. group, Ge in blood vessels combined with Bo was directly transported into brain under the enhancing effect of Borneol. We conjectured that the way Ge in Gardenia-Borneol co-compound is transported to mouse brain after i.n. administration was not through olfactory nerve-brain pathway or through the olfactory mucosa pathway. The main transport mechanism may be the blood vessel channel, which means Ge was absorbed rapidly into blood though nasal blood vessels combined with Bo, then transported into brain under the enhancing effect of Borneol, like i.v. administration [12].

The results of this research indicated that bioavailability and the brain-target effect of i.n. for Ge in Gardenia-Borneol co-compound was nearly the same as i.v. administration. Owing to the safety, convenience and cheapness of intranasal administration compared with injection treatment, developing a transnasal drug delivery system of Gardenia-Borneol co-compound is valuable, encouraging and worthy of further studies.

## Figures and Tables

**Table 1:** Main plasma pharmacokinetic parameters of the non-compartmental model in mice after i.n., i.v. and i.g. administration.

Group	Parameters				

	*C*_max_ (μg/mL)	*T*_max_ (min)	AUC_0–120_ (μg/mL·min)	MRT_0–120_ (min)	*F* (%)
i.v.	42.410 ± 6.268	-	324.88 ± 37.62	15.01 ± 1.49	100
i.n.	21.881 ± 5.398 **	1	277.39 ± 22.65 *	31.70 ± 5.68 **	85.38
i.g.	1.914 ± 0.327 **	30	93.44 ± 9.71 **	42.03 ± 6.63 **	28.76

Data are expressed as mean ± SD (*n* = 5).

* *p* < 0.05,

** *p* < 0.01 *vs.* the i.v. group.

**Table 2:** Main brain pharmacokinetic parameters of the non-compartmental model in mice after i.n., i.v. and i.g. administration.

Group	Parameters						

	*C*_max_ (ng/g)	*T*_max_ (min)	AUC_0–120_ (ng/g·min)	MRT_last_ (min)	*R*e (%)	*T*e (%)	DTI
i.v.	1476.4 ± 145.1	1	37270.5 ± 4160.6	48.3 ± 2.0	100	11.47	1
i.n.	746.7 ± 174.8 **	3	32413.6 ± 4573.9	51.2 ± 2.6	86.97	11.69	1.02
i.g.	76.2 ± 22.1 **	60 **	6440.1 ± 863.7 **	60.0 ± 3.4 **	17.28 **	6.89 **	0.60 **

Data are expressed as mean±SD (*n* = 5).

* *p* < 0.05,

** *p* < 0.01 *vs.* the i.v. group.
